# A Unique Case of Carotid Splaying by a Cervical Vagal Neurofibroma and the Role of Neuroradiology in Surgical Management

**DOI:** 10.7759/cureus.1658

**Published:** 2017-09-07

**Authors:** Sally A Itawi, Mark Buehler, Robert E Mrak, Tarek R Mansour, Yacine Medhkour, Azedine Medhkour

**Affiliations:** 1 Department of Surgery, University of Toledo Medical Center; 2 Department of Radiology, The University of Toledo Medical Center; 3 Department of Pathology, University of Toledo Medical Center

**Keywords:** carotid splaying, neurofibromatosis type 1, cervical neurofibroma, carotid body tumor, lyre sign, mra, peripheral nerve sheath tumor

## Abstract

Carotid splaying, also known as the Lyre sign, is a widening of the carotid bifurcation due to the displacement of the internal carotid artery and the external carotid artery just distal to the point of divergence. This phenomenon is classically exhibited by highly vascularized carotid body tumors and, in rare cases, by cervical sympathetic chain schwannomas. Demonstration of the Lyre sign by a cervical vagal neurofibroma, however, is a unique occurrence that has not been previously documented in the literature.

Neurofibromas are slow growing, poorly vascularized soft tissue masses and are a hallmark of the autosomal dominant genetic disorder, neurofibromatosis type 1 (NF-1). While targeted genetic therapies are evolving, management is currently dependent on a case-by-case resection of tumors with specific indications for chemo and radiation therapy. These resections rely on magnetic resonance imaging (MRI) to visualize tumor location and infiltration, but even in the setting of an established NF-1 diagnosis, additional imaging can be beneficial in ruling out more precarious tumors and optimizing surgical outcomes.

In this case, a 25-year-old female with known NF-1 presented with an enlarging cervical mass that demonstrated splaying of the left internal and external carotid arteries on MRI. Due to the typical association of the Lyre sign with carotid body tumors, magnetic resonance angiography (MRA) was crucial in guiding surgical decision making. Carotid body tumors are highly vascularized, may compress carotid branches, and carry a high risk of intraoperative bleeding. They are best visualized with MRA, which assesses carotid splaying and patency, and demonstrates vascular blushing within the tumor.

This patient's MRA demonstrated the Lyre sign, patency of all carotid vessels, and a lack of vascularity within the mass, thus lowering suspicion for a carotid body tumor. Intraoperative use of imaging results facilitated a successful resection of a soft tissue tumor with minimal blood loss and no complications. Postoperative histologic examination confirmed a neurofibroma and definitively ruled out a carotid body tumor. This case highlights the importance of utilizing MRA whenever carotid splaying is seen on MRI and supports the consideration of neurofibromas in the differential for this finding.

## Introduction

Splaying of the internal and external carotid arteries by a cervical vagal neurofibroma is a unique occurrence that has not been previously documented in the literature. This phenomenon, known as the Lyre sign, is classically exhibited by carotid body tumors and, in rare instances, by cervical schwannomas [1-2]. Carotid body tumors, or glomus tumors, originate from neural crest cells at the carotid bifurcation and are highly vascularized. Patients can present with syncope due to compromised blood flow, carotid compression, or baroreceptor overstimulation. Therefore, the gold standard for diagnosis is MRA in addition to MRI. Due to tumor vascularity, characteristic findings are punctuate vascular flow voids on T2 MRI and significant contrast enhancement on MRA [2-3]. 

Schwannomas and neurofibromas are types of peripheral nerve sheath tumors (PNSTs) and present similarly as slow-growing soft tissue masses which are best visualized on MRI. They are poorly or non-vascularized, heterogeneously bright on T2 MRI, and would not enhance on MRA. Due to their slow-growing and non-vascular nature, these tumors tend to only displace nearby vessels without compromising blood flow or causing compression [4]. Symptoms vary based on tumor size, location, and growth rate, and the most common presenting symptom of a cervical PNSTs is dysphagia [5]. These radiographic and clinical differences between PNSTs and carotid body tumors assist in the preoperative differential diagnosis; however, a final diagnosis requires histologic examination. 

While PNSTs may appear similar on gross examination, schwannomas and neurofibromas have distinct histological characteristics. Schwannomas demonstrate alternation between compact areas of high spindle cellularity (Atoni A) and less cellular, loose spongy areas (Atoni B). Neurofibromas are uniformly hypocellular with minimal mitotic activity and have elongated spindle cells with wavy nuclei and cytoplasmic processes. The background can vary between myxoid, mucinous, or “shredded carrot collagen” with alternating thick and thin collagen strands [6].

Both types of PNSTs can be managed by surgical resection on a case-by-case basis, depending on their location and the severity of neurologic deficits or cosmetic alteration. The importance of angiography in the surgical management of cervical neoplasms has been well documented in the literature and is especially useful when there is vessel displacement or compression [7-8]. There are a few documented cases of cervical sympathetic chain schwannomas causing cervical splaying similar to that seen with carotid body tumors [1, 9] and rare cases of cervical vagal neurofibromas in close proximity to the common carotid artery and its branches [7, 10]. However, there is no documentation in the literature of a vagal neurofibroma at a location that causes carotid splaying similar to that seen in either carotid body tumors or schwannomas. This unique case highlights the importance of including neurofibromas on the differential for a carotid splaying tumor and of pre- and intraoperative use of MRI and MRA images to optimize tumor resection and surgical outcomes.

## Case presentation

A 25-year-old Caucasian female with a known history of NF-1 presented to the clinic with a left cervical mass that had been present for three years but became increasingly symptomatic over the past four months. She complained of constant moderate pain and dysphagia with no alleviating or aggravating factors. She denied any cervical or extremity weakness, paresthesia, visual changes, or vertigo. Her past medical history was unremarkable. Physical examination showed a soft, tender, vertically mobile, non-pulsatile, well-defined left cervical mass with no lymphadenopathy or overlying skin changes. No focal neurological deficits were present as a result of this mass. It was noted that she had multiple café-au-lait spots on the bilateral upper extremities and back and small cutaneous neurofibromas on her back and thighs bilaterally.

**Figure 1:**
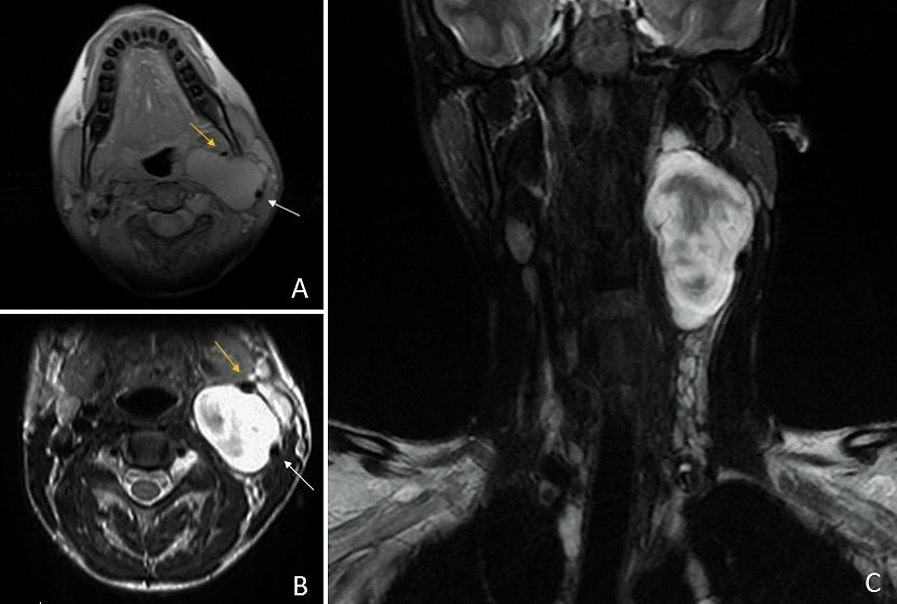
Preoperative axial and coronal cervical spine magnetic resonance imaging (MRI) Preoperative cervical MRI showing a large mass at the left carotid bifurcation with splaying of the left internal carotid artery (white arrow) and external carotid artery (yellow arrow). A) T1-weighted, axial, isointense; B) T2-weighted, axial, predominantly hyperintense; C) T2-weighted, coronal, predominantly hyperintense

**Figure 2:**
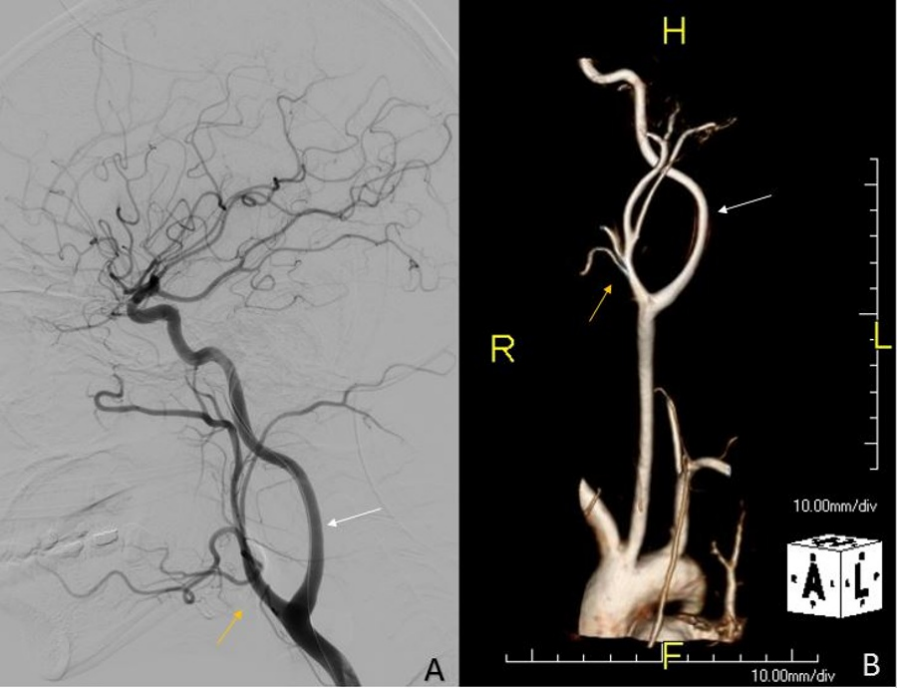
Preoperative lateral and 3D reconstructed cervical spine MRA MRA demonstrating lateral displacement of the left internal carotid artery (white arrow), anterior displacement of the left external carotid artery (yellow arrow), vessel patency, and no post-contrast enhancement at the tumor site. A) Lateral view; B) 3D reconstruction 3D: three dimensional; MRA: magnetic resonance angiography

MRI of the cervical spine revealed a 7.9 x 4.4 x 2.6 cm mass splaying the left internal and external carotid arteries. The mass was isointense on T1, predominantly hyperintense on T2, and mildly enhancing with relative homogeneity (Figure 1). No flow voids were seen within the mass. MRI of the entire spine demonstrated multiple T2 hyperintense neurofibromas within various neural foramen, paraspinal muscles, and bilateral brachial plexuses. MRA was ordered to evaluate the displaced vascular structures, the proximity of the carotid arteries to the mass, and vascularity within the mass itself. The MRA revealed normal course and caliber of the common carotid artery, and a mass splaying and displacing the left internal carotid artery anteriorly and the left external carotid artery laterally. Notably, all the vessels remained widely patent, and the mass did not show post-contrast enhancement (Figure 2). The lack of vascular blushing within the mass lowered the suspicion for a carotid body tumor and some of the concern for significant intraoperative bleeding. To optimize safety, a vascular surgeon was on reserve due to the inherent risk of operating in close proximity to major vessels. The MRI and MRA images were displayed in the room to further guide resection.

**Figure 3:**
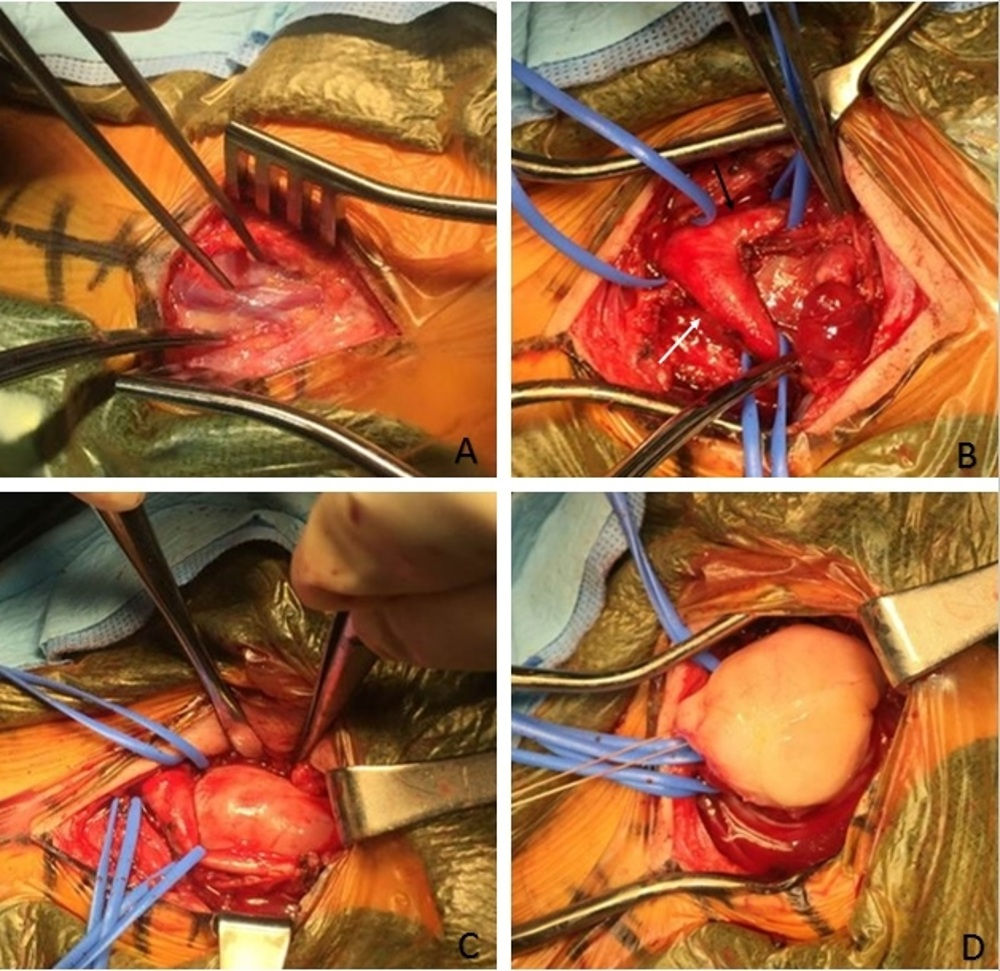
Intraoperative surgical technique and gross pathology A) Visualization of the external jugular vein; B) Visualization of the carotid bifurcation, internal carotid artery (white arrow) and external carotid artery (black arrow); C) Retraction of the carotids and visualization of the tumor; D) Resection of the tumor with a vicryl suture.

A left neck dissection with paracarotid vagal mass resection was performed by the neurosurgical team. Intraoperatively, the external jugular vein was first visualized and secured (Figure 3A), and the carotid bifurcation was carefully exposed and dissected away from the tumor (Figure 3B-3C). A vicryl stay suture was placed in the protruding aspect, and the tumor sprung to the surface with gentle retraction (Figure 3D). The tumor was grossly solid and homogenous with no vascularity, necrosis, or local adhesions or invasions.  

**Figure 4:**
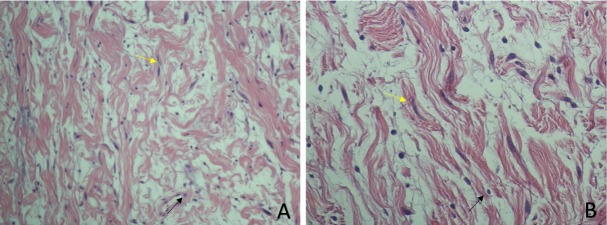
Histologic examination of tumor samples Postoperative histologic examination showing elongated spindle cells with wavy nuclei (yellow arrows), cytoplasmic inclusions (black arrows), and a “shredded carrot” collagenous background A) 20x magnification; B) 40x magnification.

Histologic examination of tumor samples revealed a hypocellular spindle cell lesion with no mitotic figures or necrotic areas. The lesion contained elongated spindle cells with marked nuclear atypia in a background with mixed collagenous and myxoid elements (Figure 4). These findings confirmed that the mass was a neurofibroma and not a schwannoma. In this case, the patient had a known diagnosis of NF-1, so immunohistochemical staining for S-100 was not performed. Postoperatively, the patient had some cervical myalgia that gradually resolved, and she was discharged home two days following the procedure with no further complications.   

## Discussion

NF-1 is associated with a myriad of multisystem findings but is hallmarked by and named for the numerous peripheral neurofibromas that develop throughout the patient’s life. These PNSTs can vary in presentation and severity, and subtypes include localized, diffuse, and plexiform. Of these, diffuse neurofibromas are the most common in the head and neck and tend to be infiltrative along neural axons but nondestructive to the surrounding structures. In this patient, the soft and slow-growing neurofibroma at her left carotid bifurcation caused the internal carotid artery and external carotid artery to splay over time but did not compromise vessel patency or cephalic blood flow. Therefore, she presented with pain and dysphagia once the tumor reached an occupancy threshold but had no symptoms that would be present in cases of compromised blood flow, such as vertigo or vision change.

MRI best visualized the location and parameters of the tumor and demonstrated the surprising finding of the Lyre sign. However, MRI alone is not reliable in distinguishing vascular characteristics of these tumors, as both neurofibromas and carotid body tumors can present as T1 hypointense and T2 hyperintense masses. Neurofibromas have more heterogeneous contrast enhancement, whereas carotid body tumors may have a “salt and pepper” appearance representing small foci of hemorrhage, but MRA findings are more specific for highly vascular carotid body tumors.

Much like the handles of a lyre, carotid splaying gives the internal and external carotid arteries a bowed appearance on MRA and is classically accompanied by blushing within the tumor space that is indicative of a vascular carotid body tumor. In this patient, the internal and external carotid arteries created a distinct outline around the tumor space but there was no vascularity terminating within it. The carotids remained strongly patent, and there was normal cerebral and ophthalmic blood flow. A Lyre sign without tumor blushing and the lack of compromise in nearby vessels increased the suspicion for a carotid splaying PNST. This was more likely to be a neurofibroma due to the patient’s known NF-1, but due to the unprecedented nature of this finding, a definitive diagnosis was made based on histologic results. 

Intraoperatively, MRI and MRA results were utilized to locate the displaced vasculature and expose the carotid bifurcation and its branches. Due to the lack of vascular supply to the tumor and its lack of local adhesions, the vessels were safely retracted and secured away from the plane of resection with minimal resistance and no blood loss. The tumor began protruding on its own and was easily retractable with a vicryl suture and application of mild pressure. The mass remained intact throughout retraction and demonstrated no vascularity or adhesions on its posterior aspect. On gross examination, the tumor appeared solid and uniformly pale yellow in color, which reaffirmed the diagnosis of a PNST. Overall, it had no vascular recruitment and only occupied space within the carotid sheath without adhering to nearby soft tissues. While the patient’s presentation and operative findings were most consistent with a neurofibroma, the diagnosis was affirmed with histologic findings of elongated spindle cells, wavy nuclei, and a collagenous background.

## Conclusions

While the Lyre sign, or splaying of the internal carotid artery and external carotid artery at the carotid bifurcation, is classically associated with carotid body tumors and, in some cases, with cervical schwannomas, this unique case demonstrates the occurrence of this phenomenon with a peripheral neurofibroma. Vagal nerve neurofibromas should be included in the differential diagnosis for carotid splaying tumors, especially when MRI and MRA findings are consistent with a PNST. When resection is in question, preoperative MRA is imperative to rule out a highly vascularized carotid body tumor, ensure vessel patency, and support surgical interventions. Intraoperative use of imaging results to isolate and retract the carotids allows for a successful resection with minimal blood loss. Postoperative histology of the PNST differentiates a schwannoma from a neurofibroma. Even in a patient with known NF-1, the rare finding of the Lyre sign on MRI warrants a thorough preoperative investigation with MRA and postoperative histologic confirmation of a carotid-splaying neurofibroma. 
